# The *TOMM40 ‘523’* polymorphism in disease risk and age of symptom onset in two independent cohorts of Parkinson’s disease

**DOI:** 10.1038/s41598-021-85510-0

**Published:** 2021-03-18

**Authors:** Megan C. Bakeberg, Madison E. Hoes, Anastazja M. Gorecki, Frances Theunissen, Abigail L. Pfaff, Jade E. Kenna, Kai Plunkett, Sulev Kõks, P. Anthony Akkari, Frank L. Mastaglia, Ryan S. Anderton

**Affiliations:** 1 Perron Institute for Neurological and Translational Science, Nedlands, WA Australia; 2 Centre for Neuromuscular and Neurological Disorders, University of Western Australia, Nedlands, WA Australia; 3 School of Biological Sciences, University of Western Australia, Crawley, WA Australia; 4 The Centre for Molecular Medicine and Innovative Therapeutics, Murdoch University, Murdoch, WA Australia; 5 Institute for Health Research and School of Health Sciences, University of Notre Dame Australia, 19 Mouat Street, Fremantle, WA 6959 Australia

**Keywords:** Neurodegenerative diseases, Parkinson's disease, Genetics research, Risk factors, Disease genetics, Genetic markers

## Abstract

Abnormal mitochondrial function is a key process in the pathogenesis of Parkinson’s disease (PD). The central pore-forming protein TOM40 of the mitochondria is encoded by the translocase of outer mitochondrial membrane 40 homologue gene (*TOMM40*). The highly variant *‘523’* poly-T repeat is associated with age-related cognitive decline and age of onset in Alzheimer’s disease, but whether it plays a role in modifying the risk or clinical course of PD it yet to be elucidated. The *TOMM40 ‘523’* allele length was determined in 634 people with PD and 422 healthy controls from an Australian cohort and the Parkinson’s Progression Markers Initiative (PPMI) cohort, using polymerase chain reaction or whole genome sequencing analysis. Genotype and allele frequencies of *TOMM40 ‘523’* and *APOE* ε did not differ significantly between the cohorts. Analyses revealed *TOMM40 ‘523’* allele groups were not associated with disease risk, while considering *APOE* ε genotype. Regression analyses revealed the *TOMM40* S/S genotype was associated with a significantly later age of symptom onset in the PPMI PD cohort, but not after correction for covariates, or in the Australian cohort. Whilst variation in the *TOMM40 ‘523’* polymorphism was not associated with PD risk, the possibility that it may be a modifying factor for age of symptom onset warrants further investigation in other PD populations.

## Introduction

Parkinson’s disease (PD) is increasingly known as a multifaceted neurodegenerative disorder with a heterogeneous and burdensome symptom presentation and progression. Abnormal or deficient mitochondrial functioning is widely implicated as a key process in the selective neuronal death and pathogenesis of PD. Mitochondrial dysfunction, resulting in a loss of electron transport chain (ETC) efficiency or decline in ATP-synthesising capacity, appears to elicit dopaminergic cell death via a number of mechanisms, including reactive oxygen species(ROS)—generation, impaired ATP production and disrupted calcium homeostasis^1,2^. The pivotal role of mitochondrial dysfunction in PD is supported by several parkinsonism-causing toxins, genetic mutations and retrotransposon insertions^3,4^, which specifically impair mitochondrial function. Insights from these toxins and mutations imply that mitochondrial dysfunction in the pathogenesis of PD can arise from a wide array of biological processes, such as bioenergetic disturbances, nuclear and mitochondrial DNA mutations, impaired fusion and fission, defective mitophagy, and abnormal morphology and size^5^. For example, a significant cause of mitochondrial dysfunction in PD is the inhibition of mitochondrial complex I, an ETC defect that leads to severe oxidative stress and ROS- and caspase- mediated dopaminergic cell death^5^. Notably, the latter impairment is a major pathological feature of PD induced by familial PTEN-induced kinase 1 (*PINK1*), alpha-synuclein (*SNCA*) and Daisuke-Junko-1 (*DJ-1*) gene mutations or the toxin 1-methyl-4-phenyl-1,2,3,6- tetrahydropyridine (MPTP)^3^. Thus, mitochondrial dysfunction is thought to cause neuronal stress and degeneration, eventuating in this neurodegenerative disease, PD.

The translocase of outer mitochondrial membrane 40 homologue (*TOMM40)* gene encodes the pore-forming subunit (TOM40) of the protein-transport channels in the mitochondrial outer membrane, thus playing a fundamental role in mitochondrial functioning^6^. The TOM40 protein facilitates the import of approximately 1500 externally synthesised proteins and peptides into the mitochondria, and plays a key role in the mitophagy degradation pathway^7,8^. Altered or abnormally functioning TOM40, mediated by genetic changes or atypical protein expression, is thought to contribute to mitochondrial dysfunction and protein accumulation in Alzheimer’s disease (AD) and PD^8^. This may occur through different mechanisms: (1) mitochondrial import impairment, which may prevent essential proteins and peptides from reaching their designated mitochondrial targets, or allow unwanted and mutant proteins to aggregate in the mitochondria; or (2) mitophagy disruption, which may enable damaged and malfunctioning mitochondria to accumulate. Given evidence for mitochondrial dysfunction in PD, genetic variants affecting *TOMM40* expression may have risk and disease-modifying effects.

Downstream of *TOMM40* is apolipoprotein E (*APOE*), which is in a region of strong linkage disequilibrium (LD), or non-random association, with *TOMM40* variation^9–11^. Though the extent to which common variants in *TOMM40* can have *APOE*-independent effects on disease risk or disease-modifying effects remains uncertain, polymorphisms in *TOMM40* have been independently associated with a range of primarily cognition-based neurodegenerative diseases, including AD, frontotemporal dementia and dementia with Lewy bodies, as well as non-pathological ageing^8,12–15^. One of these variants is situated in intron 6 of *TOMM40* and is a homopolymer of repeating thymine (T) nucleotides known as rs10524523 or *‘523’*^9,16^. The *‘523’* variant is polymorphic, with lengths that vary from 11 to 39 T residues in Caucasians^9,17^. To better categorise and describe this diverse structural variant, three allelic groups for the homopolymer have been established previously, according to the number of constituent T residues: ‘Short’ (S, T ≤ 19), ‘Long’ (L, 20 ≤ T ≤ 29) and ‘Very Long’ (T ≥ 30)^9,17,18^. Although the functional effects of the *‘523’* variation are mostly unknown, increasing length of the poly-T repeat is thought to increase *TOMM40* expression^19,20^. Therefore, the effect of the *TOMM40 ‘523’* variant and altered TOM40 protein levels may contribute to mitochondrial dysfunction in PD^16,21^. While a vast number of published studies report conflicting roles of the various *TOMM40 ‘523’* variant lengths in AD and ageing populations^22–26^, there have only been two previous studies within PD cohorts with differing findings. The first was a study of a relatively large Polish PD cohort which found no significant associations between *TOMM40 ‘523’* and PD risk or age of onset^27^. Subsequently, a significant overrepresentation of the L/VL *‘523’* genotype was reported in a Swedish PD cohort compared to healthy controls, though it should be noted that these findings are yet to be published in full^28^. Thus, with conflicting findings in these two European cohorts, further investigation in other populations is required.

Due to the essential role of *TOMM40* in mitochondrial import and mitophagy, both plausible sources of PD-causing mitochondrial dysfunction, the *‘523’* variant in *TOMM40* is therefore a potential risk factor and disease modifier in PD. This study investigated the distribution of *‘523’* alleles and their association with disease risk and age of symptom onset in two independent PD cohorts, one Australian and one international (the Parkinson’s Progression Markers Initiative, PPMI), using PCR-based and whole genome sequencing approaches.

## Methods

### Cohorts

The first cohort comprised of 235 home-based people with PD (PwP) and 231 healthy controls of European heritage from the Perron Institute for Neurological and Translational Science PD Database, as previously reported^29^. Clinical and demographic data, including the age of symptom onset, were recorded in the database. All PwP were examined by a movement disorder neurologist prior to inclusion in the study for verification of the diagnosis in accordance with the UK Brain Bank criteria for idiopathic PD and reported no family history of PD^30^, while healthy controls were confirmed to have no history of any neurological disorders. This study was approved by the Sir Charles Gairdner Hospital Human Research and Ethics Committee (Approval number 2006/073). Written informed consent was obtained from all participants, in accordance with the Australian National Health and Medical Research Council research guidelines.

The second cohort was derived from the international Parkinson’s Progression Markers Initiative (PPMI) database (available at http://www.ppmi-info.org/data). This cohort comprised of 368 PwP and 172 healthy controls, and only individuals of European heritage were included in order to reflect the composition of the Australian cohort.

### DNA extraction

In the Australian cohort, DNA was extracted from either blood samples or buccal swabs. Participant buccal samples were collected by a trained researcher using Isohelix DNA/RNA Buccal Swabs (Cell Projects Ltd, Kent, U.K.) and stored until DNA extraction. Alternatively, blood was collected from the medial cubital vein. DNA was extracted and purified from these samples using QIAamp DNA mini kits (Qiagen Pty LTD., Victoria, Australia), according to the manufacturer’s protocol. DNA concentration was determined using absorbance readings calculated by a NanoDrop One Microvolume UV–Vis spectrophotometer (Thermo Fisher Scientific Australia Pty LTD., Victoria, Australia).

### Genotyping of *TOMM40 ‘523’* using PCR and fragment analysis

PCR-amplification of the *‘523’* variant in the Australian cohort was completed using fluorescently labelled primers, as previously described^19^. The forward primer sequence was 5′-/6-FAM/-TGCTGACCTCAAGCTGTCCTC-3′ and the reverse primer was 5′-GAGGCTGAGAAGGGAGGATT-3′, synthesized by Integrated DNA Technologies Pty Ltd (IDT, Iowa, USA). Endpoint PCR was performed using 5 μL of 5 × MyFi Reaction Buffer (including 1 mM dNTPs and 3 mM MgCl2; Bioline, NSW, Australia), 1 μL of MyFi DNA Polymerase (Bioline), 0.2 μL of each forward and reverse primer (20 μM) (IDT), 0.25 μL of 1% dimethylsulfoxide (DMSO; Sigma), 50 ng of genomic DNA, and 11.35 μL of dH2O (Baxter Healthcare); to a final volume of 25 µL. Optimised PCR conditions were as follows: 1 cycle at 94 °C for 3 min, 27 cycles at 94 °C for 15 s, annealing at 65 °C for 20 s and extension at 70 °C for 30 s, and 1 cycle at 70 °C for 5 min. Applied Biosystems SimpliAmp Thermal Cyclers (Thermo Fisher Scientific, MA, USA) were used for endpoint PCR cycling. Post PCR products were stored at 4 °C until capillary fragment separation, which was conducted by the Australian Genome Research Facility (AGRF, WA, Australia). Electropherograms were analysed using Peak Scanner Software (v1.0; Thermo Fisher Scientific). *‘523’* allele lengths were determined according to a previously established method by Linnertz et al.^19^. Briefly, the highest intensity peak(s) in each peak cluster between 160–190 bp were identified and sized, and 150 bp (accounting for flanking regions and primers) was subtracted from the peak sizes to determine poly-T allele lengths. Alleles were grouped using the convention established by Roses et al.^9^: Short (S, T ≤ 19), Long (L, 20 ≤ T ≤ 29) and Very Long (VL, T ≥ 30).

### Genotyping of TOMM40 *‘523’* using Whole Genome Sequencing

Whole genome sequencing (WGS) data were obtained from the PPMI database (available at http://www.ppmi-info.org/data) in binary alignment map (BAM) format that had been aligned to the human reference genome GRCh38 using the Burrows-Wheeler transform alignment algorithm^31^. Resultant BAM files were analysed using the Integrative Genomics Viewer^32^ in order to calculate the length of the poly-T repeat, as previously demonstrated^33^. Alleles were grouped using the convention established by Roses et al.^9^: Short (S, T ≤ 19), Long (L, 20 ≤ T ≤ 29) and Very Long (VL, T ≥ 30).

### APOE ε genotyping

Genotyping of *APOE* ε were determined using the single nucleotide polymorphism (SNP) and PCR-restriction fragment length polymorphism (PCR–RFLP) analyses. *APOE* ε2/ε3/ε4 genotypes were determined by sequencing two SNPs (rs429358 and rs7412) using the MassARRAY system (Agena, Biosciences) at the Australian Genome Research Facility (AGRF; Queensland, Australia)^34^. For PCR–RFLP analyses, endpoint PCR reactions were prepared to a final volume of 10 µl using primer sequences previously described^35^. Reactions contained 7.2 µl dH_2_O (Baxter Healthcare, NSW, Australia), 2 µl MyFi reaction buffer (Bioline, NSW, Australia,), 0.05 µl MyFi DNA polymerase (Bioline, NSW, Australia), 0.375 µl forward and, 0.375 µl reverse primer (Integrated DNA Technologies, Iowa, USA) at 200 ng/µl, and 25 ng DNA. The PCR amplification protocol followed, being an initial hold temperature of 95 °C for 4 min 30 s and 35 cycles of denaturation at 95 °C for 30 s, annealing 60 °C for 30 s and extension at 72 °C for 1 min 30 s. Restriction enzyme reactions were prepared to a final volume of 20 µl, containing 6.3 µl dH_2_O (Baxter Healthcare, NSW, Australia), 0.2 µl acetylated BSA (Promega, WI, USA), 2 µl C buffer (Promega, WI, USA), 0.5 µl *Hhal* (Promega, WI, USA) and 10 µl PCR product. Reactions were incubated for 4 h at 37 °C prior to polyacrylamide gel fractionation. Restriction enzyme digested products were fractionated on 12% (w/v) 29:1 polyacrylamide gel (BioRad, CA, USA) in 1 × TBE. Electrophoresis fragment separation was performed at 100 V for 3 h on the DCode Universal Mutation Detection System (BioRad, CA, USA). Gels were stained in 1 × TBE containing SYBR Gold nucleic acid gel stain (Thermo Fisher Scientific, MA, USA) for 4 min before visualization using a BioRad Chemidoc MP Imaging System. *APOE* ε genotype data relating to the PPMI cohort was obtained from the online database (available at www.ppmi-info.org/data).

### Statistical methods

The Australian and PPMI cohorts were analysed separately, and together, using IBM-SPSS software (version 26, IBM Corporation). A significant nominal *p*-value of < 0.05 was employed for all statistical tests. Variables were described using mean and standard deviation (in brackets, SD), or frequency and percent (in brackets, %), as appropriate. Normality was assessed and subsequent clinical characteristics were analysed using Independent Samples T-Test, Mann–Whitney *U*, or Chi-square, as appropriate. For cross-sectional analysis, Chi-square, stratified Mantel–Haenszel tests and binary logistic regression models were used to evaluate the association between *TOMM40 ‘523’* genotypes and risk of PD in the Australian and PPMI cohorts. Binary logistical regression models were run both with and without correction for the *APOE* ε4 status (being grouped as zero, one or two ε4 allele(s)) and patient sex. Analysis was also run considering all combinations of *‘523’* length category and *APOE* ε genotype, to examine for interactive effects without a priori assumptions. The aforementioned analyses were performed separately in the PPMI and Australian cohorts, and after combination of the cohorts. Following this, binary logistic models were carried out when considering populations of *APOE* ε3/ε3 carriers only, as previously examined in this fashion and stated as a requirement for replication studies^13^.

Generalised linear models (GLMs) were also constructed in order to study the interaction of *TOMM40* on age of disease onset, correcting for *APOE* ε allele status and sex. Again, GLMs were carried out when considering populations of *APOE* ε3/ε3 carriers only. Residual plots were examined for all models and no violations were noted. Correction for multiple comparisons was conducted using Bonferroni pairwise comparisons, where appropriate.

Subsequently, the load combination of *TOMM40 ‘523’* S allele carrier status (being S/S genotype, carriage of one S allele, and non-carriage of the S allele) and *APOE* ε4 status (the genotype ε4/ε4, carriage of one ε4 allele and non-carriage of the ε4 allele) were combined to produce 5 groups. Mean comparisons were then analysed using the Kruskal–Wallis one-way analysis of variance and univariate GLMs. This analysis was repeated in the combinations of *TOMM40 ‘523’* S allele carrier status and *APOE* ε2 status, and *TOMM40 ‘523’* VL allele carrier status and *APOE* ε4 status.

Finally, Kaplan–Meier curves for age at PD symptom onset were estimated, stratified by *TOMM40 ‘523’* genotype as well as both the *TOMM40 ‘523’* and *APOE* genotype. To compare the survival curves, the log rank test was applied, placing weight on longer survival periods^36,37^. Allelic stratification by *TOMM40 ‘523’* was also run using Kaplan–Meier analysis. Additionally, all distributions of ages at onset adjusting for sex were compared via Cox proportional hazard regression models.

## Results

### Cohort information

A summary of the cohorts included in this study is presented in Table 1. The Australian cohort (n = 466) consisted of 231 controls and 235 PwP. Both the controls and PwP of this cohort consisted of more males (63.6% and 63.8%, respectively), and were aged 63.9 and 65.9 years, respectively. The PPMI cohort (n = 540) was comprised of 172 controls and 368 PwP. This cohort was similarly dominated by males (65.7% of controls, 65.5% PwP), with controls having a mean age of 61.6 years and those with PD having a mean age of 61.8 years.

**Table 1:** Summary of the independent and combined cohort demographic information.

	Australian cohort		PPMI cohort		Combined cohort	
	PD	Control	PD	Control	PD	Control
*N*	235	231	368	172	603	403
Male	150 (63.8)	147 (63.6)	241 (65.5)	113 (65.7)	391 (64.8)	260 (64.5)
Female	85 (36.2)	84 (36.4)	127 (34.5)	59 (34.3)	212 (35.2)	143 (35.5)
Age (years)	65.9 (9.4)	63.9 (12.5)	61.8 (9.6)	61.6 (10.5)	63.3 (9.7)	62.9 (11.7)
Symptom onset age (years)	57.3 (10.6)	-	59.9 (9.7)	-	58.9 (10.1)	-

Data presented as Mean (SD) or n (%).

PPMI, Parkinson’s Progression Markers Initiative; PD, Parkinson’s disease.

### *TOMM40 ‘523’* and *APOE* ε genotyping and distribution

**Figure 1:**
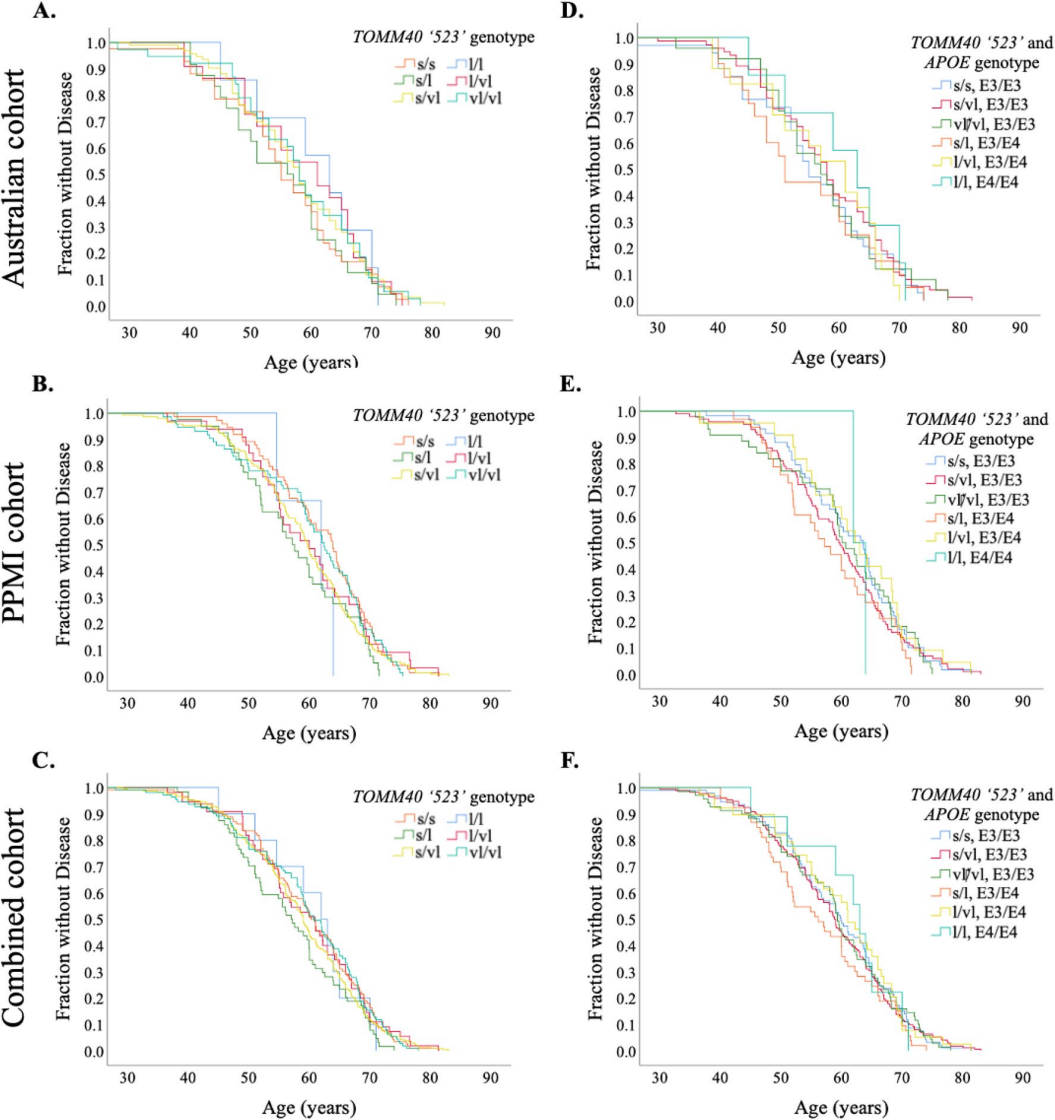
Age of onset of PD symptom curves for *TOMM40 ‘523’* genotypes. (**A**–**C**) indicate the age at which symptoms of PD began when stratified by *TOMM40 ‘523’* genotype, as demonstrated by Kaplan–Meier curves. (**C**–**E**) indicate the age at which symptoms of PD began when stratified by *TOMM40 ‘523’* and *APOE* genotype, as demonstrated by Kaplan–Meier curves. PPMI, Parkinson’s Progression Markers Initiative; S/S, short/short; S/L, short/long; S/VL, short/very long; L/L, long/long; L/VL, long/very long; VL/VL, very long/very long; S, short; L, long; VL, very long; ε2/ε2, Apolipoprotein epsilon 2/epsilon 2; ε2/ε3, Apolipoprotein epsilon 2/epsilon 3; ε2/ε4, Apolipoprotein epsilon 2/epsilon 4; ε3/ε3, Apolipoprotein epsilon 3/epsilon 3; ε3/ε4, Apolipoprotein epsilon 3/epsilon 4; ε4/ε4, Apolipoprotein epsilon 4/epsilon 4.

In the Australian cohort, *TOMM40 ‘523’* genotyping was obtained following optimisation of a PCR-based assay, with representative electropherograms presented in Supplementary Fig. 1. Genotyping of the *TOMM40 ‘523’* variant in the PPMI cohort was completed using a WGS method, previously reported as an alternative method to fragment analysis and Sanger sequencing^33^. The distribution of the *‘523’* alleles in PwP compared to healthy controls for both cohorts are presented in Fig. 1. Following prior literature, allele sizes were used to bin samples into Short (S, T ≤ 19), Long (L, 20 ≤ T ≤ 29) and Very Long (VL, T ≥ 30) groups^9^ (Supplementary Table 1). *APOE* ε genotypes of the Australian cohort were further validated using PCR–RFLP, with representative samples ε2/ε3, ε2/ε4, ε3/ε3 and ε3/ε4 presented in Supplementary Fig. 2. *APOE* ε genotype and allele groupings for both cohorts exhibited similar distributions, however there was an absence of individuals with the ε2/ε2 genotype in the Australian cohort (see Supplementary Table 2).

Both groups consisted of individuals with self-reported of European heritage, though country of origin information was not available for all cases. To determine whether cohorts could be appropriately combined, Levene's Test for Equality of Variances was used to compare the *‘523’* distribution and no significant difference was observable (F = 1.772, *p* = 0.183). Each distribution was also compared to distributions on Webstr database, similarities were seen between the current study cohorts and both the Gtex (predominantly European self-reported ancestry) and 1000 genomes European distributions. Thus, the current cohort were considered reflective of a population of European descent and the two cohorts could be combined to increase the sample size and power to detect *‘523’* effect.

### *TOMM40 ‘523’* not associated with PD risk

As there were no significant differences in *TOMM40* allele distributions between cohorts, case–control analyses were completed in both individual cohorts and in a combined cohort. When examined genotypically, the *TOMM40 ‘523’* variant was not significantly associated with the risk of PD in either the Australian cohort (χ2 = 1.455, *p* = 0.918), or the PPMI cohort (χ2 = 1.806, *p* = 0.875), or when cohorts were combined (χ2 = 3.471, *p* = 0.628). Moreover, when combined, allelic distribution of the S (44.6% Vs 44.7%, Supplementary Table 1), L (11.8% Vs 13.9%, Supplementary Table 1) and VL (43.6% Vs 41.4%, Supplementary Table 1) binned groups was not significantly different between PD and controls (χ2 = 0.003, *p* = 0.953; χ2 = 0.723, *p* = 0.395; χ2 = 0.149, *p* = 0.699; respectively). Analysis considering all combinations of *‘523’* genotypes and *APOE* ε genotypes also failed to show any significant differences between PD and control groups. As with prior findings, analysis utilising stratified Mantel–Haenszel tests did not find significant differences.

Binary logistic regression models were analysed comparing each genotype or allele to all other genotypes or alleles (Table 2). Genotypically, naïve models exhibited a lack of significant effect on PD risk, both in each individual cohort and when combined. Furthermore, models were constructed taking *APOE* ε4 status and patient sex into account, which again exhibited no significant association between *TOMM40 ‘523’* genotype and PD risk. Models considering all combinations of *‘523’* and *APOE* ε genotypes also returned non-significant results. Binary logistic regression models were used subsequently to assess this association allelically. Neither naïve nor covariate corrected models, in either cohort or the combined cohort, exhibited a significant allelic association between *TOMM40 ‘523’* and disease risk (Table 2). Finally, all models were repeated in the sub-cohort of *APOE* ε3/ε3 carriers (Supplementary Table 3), and again failed to show any significant associations between *TOMM40 ‘523’* alleles or genotypes and risk of PD.

**Table 2:** Corrected regression models evaluating the association between *‘523’* genotype and disease risk in the two PD cohorts.

TOMM40 ‘523’	Australian cohort (n = 466)				PPMI cohort (n = 540)				Combined cohort (n = 1006)			
	Naïve^+^		Corrected^#^		Naïve^+^		Corrected^#^		Naïve^+^		Corrected^#^	
	OR (95% CI)	*p*	OR (95% CI)	*p*	OR (95% CI)	*p*	OR (95% CI)	*p*	OR (95% CI)	*p*	OR (95% CI)	*p*
S/S	1.112 (0.697–1.772)	.656	0.994 (0.608–1.624)	.980	0.943 (0.597–1.490)	.802	0.968 (0.588–1.593)	.899	1.008 (0.732–1.387)	.963	0.985 (0.700–1.387)	.932
S/L	1.112 (0.624–1.980)	.719	1.167 (0.597–2.278)	.652	0.958 (0.532–1.726)	.888	0.978 (0.496–1.928)	.950	1.040 (0.695–1.557)	.847	1.061 (0.666–1.690)	.804
S/VL	0.941 (0.650–1.363)	.748	0.876 (0.570–1.345)	.545	1.006 (0.694–1.456)	.977	0.971 (0.641–1.472)	.891	0.979 (0.756–1.267)	.871	0.940 (0.702–1.257)	.675
L/L	1.018 (0.376–2.759)	.972	0.760 (0.210–2.753)	.676	1.431 (0.237–8.646)	.696	2.125 (0.261–17.264)	.481	1.369 (0.576–3.255)	.477	1.328 (0.469–3.758)	.593
L/VL	1.228 (0.674–2.236)	.502	1.265 (0.632–2.533)	.506	1.412 (0.791–2.521)	.244	1.315 (0.629–2.750)	.467	1.315 (0.872–1.985)	.192	1.289 (0.786–2.116)	.315
VL/VL	0.793 (0.484–1.300)	.358	1.022 (0.595–1.756)	.936	0.854 (0.534–1.366)	.510	0.925 (0.562–1.523)	.759	0.801 (0.573–1.121)	.195	0.915 (0.640–1.307)	.915
S	1.038 (0.698–1.543)	.855	0.898 (0.573–1.407)	.639	0.945 (0.638–1.401)	.778	0.938 (0.610–1.441)	.769	0.992 (0.753–1.306)	.953	0.938 (0.693–1.270)	.678
L	1.174 (0.771–1.788)	.454	2.029 (0.712–2.779)	.185	1.076 (0.704–1.644)	.736	1.025 (0.419–2.507)	.957	1.135 (0.847–1.522)	.395	1.378 (0.713–2.661)	.340
VL	0.885 (0.602–1.301)	.534	0.973 (0.640–1.480)	.900	1.047 (0.708–1.547)	.819	1.002 (0.663–1.514)	.992	0.948 (0.725–1.241)	.699	0.967 (0.726–1.288)	.820

^+^Data taken from Binary Logistic Regression models without correction for covariates.

^#^Data taken from Binary Logistic Regression models with correction for *APOE* ε4 load and patient sex.

*P* and OR (95% CI) values are calculated for the comparison to all other genotypes or alleles.

PPMI, Parkinson’s Progression Markers Initiative; PD, Parkinson’s disease; OR, Odd’s Ratio*;* CI, confidence interval; *p*, statistical significance (*p* value); S/S, short/short; S/L, short/long; S/VL, short/very long; L/L, long/long; L/VL, long/very long; VL/VL, very long/very long; S, short; L, long; VL, very long.

### TOMM40 variant and age of disease onset

To investigate the influence of *TOMM40 ‘523’* on age of disease onset, GLMs were constructed, with data from naïve and corrected models reported in Table 3. Within the Australian cohort, no significant association between *TOMM40 ‘523’* alleles or genotypes and age of PD symptom onset was observed. However, in the PPMI cohort, carriage of the *TOMM40 ‘523’* S/S genotype was associated with a 2.58 year later age of symptom onset (*p* = 0.040). This difference was not significant after controlling for *APOE* ε4 load and sex in multivariable GLMs, although a trend for later age of onset was still observable (1.970 years, *p* = 0.157). It is important to note, however, that there was a lack of co-carriage of the S/S genotype with the *APOE* ε4/ε4 genotype in this cohort (see Supplementary Table 4) and that *APOE* ε4/ε4 homozygotes had a mean age of symptom onset almost 4 years earlier than other genotypes, although this was not statistically significant (*p* = *0.3*14). Thus, significance may have been lost due to opposing directionalities of these two genotypes on age of symptom onset. Carriage of the S/S genotype was also associated with a 2.69 year later age of PD symptom onset in the PPMI cohort (*p* = 0.032) after correcting for *APOE* ε2 load and sex. However, when adjusting for multiple comparisons, model-derived estimated means revealed the S/S genotype did not result in a significantly different age of PD symptom onset when compared to individual genotypes (*p* = 0.326).

**Table 3:** Generalised linear model investigating association between *TOMM40 ‘523’* and age of PD symptom onset in the Australian and PPMI cohorts.

TOMM40 ‘523’	Australian cohort (n = 226)				PPMI cohort (n = 368)				Combined cohort (n = 594)			
	Naïve^+^		Corrected^#^		Naïve^+^		Corrected^#^		Naïve^+^		Corrected^#^	
	β-CoE	*p*	β-CoE	*p*	β-CoE	*p*	β-CoE	*p*	β-CoE	*p*	β-CoE	*p*
S/S	−2.008	.267	−1.388	.469	**2.580**	**.040**	1.970	.157	0.957	.361	0.728	.525
S/L	−2.222	.331	−1.935	.538	−2.460	.129	−2.759	.181	−2.355	.078	−2.464	.160
S/VL	0.816	.569	1.354	.424	−1.228	.235	−1.301	.259	−0.487	.566	−0.455	.638
L/L	3.399	.403	4.142	.715	0.312	.956	10.474	.214	1.602	.620	8.047	.156
L/VL	1.417	.551	1.717	.583	0.131	.941	1.967	.364	0.587	.682	1.131	.520
VL/VL	0.646	.732	−0.482	.821	0.653	.607	0.082	.952	0.770	.470	0.159	.892
S	−1.261	.416	0.088	.960	−0.561	.613	−0.903	.458	−0.838	.358	−0.631	.536
L	0.087	.958	0.644	.932	−1.149	.338	0.647	.812	−0.687	.485	0.375	.887
VL	1.887	.209	1.480	.370	−0.824	.448	−0.689	.552	0.227	.799	−0.004	.997

^+^Data taken from GLM without correction for covariates.

^#^Data taken from GLMs corrected for *APOE* ε4 load and patient sex.

*P* and β-CoE values are calculated for the comparison to all other genotypes or alleles.

PPMI, Parkinson’s Progression Markers Initiative; PD, Parkinson’s disease; β-CoE*,* β-Coefficient*; p*, statistical significance (*p* value); S/S, short/short; S/L, short/long; S/VL, short/very long; L/L, long/long; L/VL, long/very long; VL/VL, very long/very long; S, short; L, long; VL, very long; GLM, generalised linear model.

Subsequent analysis of the PPMI cohort exhibited no other statistically significant associations (at the 0.05 level of significance), either allelically or genotypically. Furthermore, when the cohorts were analysed as a combined cohort, no significant associations were exhibited between *TOMM40 ‘523’* and age of PD symptom onset (Table 3). Following this, all models were also repeated in the large sub-cohort consisting of only *APOE* ε3/ε3 homozygotes (Supplementary Table 5), none of which revealed a significant association between *TOMM40 ‘523’* and age of symptom onset. Finally, when considering the load combination of the *TOMM40 ‘523’* S allele and *APOE* ε4 or *APOE* ε2, no significant differences were found in the mean age of symptom onset between groups in either cohort or in the combined cohort according to the copy number of these alleles.

Kaplan–Meier curves for the age at which symptoms of PD were first reported were stratified by *TOMM40 ‘523’* genotype, in the Australian cohort (Fig. 1A), the PPMI cohort (Fig. 1B) and the combined cohort (Fig. 1C). No or statistically significant or notable stratification was noted (*p* = 0.782, *p* = 0.321, *p* = 0.399), respectively. Subsequently, Kaplan–Meier curves for the age at which symptoms of PD began were analysed, stratified by *TOMM40 ‘523’* and *APOE* genotype as in Roses et al.^13^. Again, neither the Australian cohort (*p* = *0.5*50, Fig. 1D), or the PPMI cohort (*p* = 0.867, Fig. 1E), or the combined cohort (*p* = 0.660, Fig. 1F) exhibited statistically stratified ages of PD symptom onset. Kaplan–Meier analysis based on *TOMM40* *‘523’* allelic stratification also failed to produce significant results. Finally, all analyses completed using the Kaplan–Meier method were re-run using Cox proportional hazard regression models, which adjusted for sex, and no significant results were noted.

## Discussion

The current study aimed to investigate the *TOMM40 ‘523’* structural variant as a potential PD risk factor and modifier of the age at symptom onset. The distribution of *‘523’* poly-T alleles did not vary between PwP and healthy controls, or in the two independent cohorts examined. The similarity in *‘523’* allelic distribution between the PPMI and Australian cohorts is worth noting as this study utilised two different approaches in the calling of the *TOMM40 ‘523’* variant, establishing a PCR-based and a WGS-based assay for use in the Australian and PPMI cohorts, respectively. Assay development for the *TOMM40 ‘523’* variant is generally considered to be difficult, as poly-T variants are challenging to sequence^13,19^. Despite optimisation within this study, PCR stuttering was observed in the PCR-based assay similar to previous reports^13,19^, which is a standard complication when amplifying repetitive genomic sequences, and highlights the need for further optimisation of the assay. By comparison, calling from WGS files revealed a similar distribution as reported previously^33^. While it has been presented as having fewer challenges in optimisation and sequencing, calling of *‘523’* variants was an arduous process and the aforementioned study has stated that the correlation between *‘523’* calling by WGS and PCR-based methods decreases with increasing size of the *‘523’* allele^33^.

The present study then examined whether the *TOMM40 ‘523’* variant is implicated in PD risk and found no association between carriage of allelic variants and the risk of developing PD. When binned, genotypic and allelic frequencies appeared to be similar between controls and PwP in both independent cohorts, and when the cohorts were combined. This agrees with previous work in a Polish PD cohort which showed no association of *‘523’* alleles, genotypes or haplotypes with the risk of PD or age of symptom onset^27^. In contrast, in a Swedish population, a higher frequency of the L/VL genotype was observed in PD patients compared to controls^28^, though these findings are yet to be published in full. Given that these studies were conducted in different ethnic groups, and that* ‘523’* allele frequencies are ethnic-specific^18^, the contradictory findings in the studies to date may indicate that* ‘523’* is a risk factor in some populations, but not in others. As this is only the third study to investigate the role of *TOMM40 ‘523’* in PD risk, further studies should be performed in other ethnically diverse populations.

As mitochondrial involvement in PD is thought to be a key contributor to neuronal dysfunction and degeneration, it is plausible that a genetic variant that modulates mitochondrial function could modify age of symptom onset. Furthermore, AD and PD share a number of clinical, pathological and molecular features including toxic protein accumulation and mitochondrial dysfunction in the form of respiratory chain defects, oxidative stress, mitochondrial DNA damage and morphological abnormalities^5,38,39^. Previous studies have implicated *TOMM40 ‘523’* length in the age of onset of AD, particularly in carriers of the *APOE* ε3 allele^9,22^, but mixed findings exist. For instance, although Roses et al.^9^ reported that longer *‘523’* allele lengths were associated with risk of AD and earlier age of onset, the initial findings were not replicated in other populations^22,40^. Such varied findings may be a result of varied methodology and varied consideration of the influence of the *APOE* ε locus^13^, which is well-established as the strongest genetic predictor of AD. Most studies to date report no association of *APOE* ε variation with susceptibility to PD^41,42^, as was also the case in the current study. While the present findings did not show any positive evidence of interactive effects between *TOMM40 ‘523’* and *APOE* ε genotypes in relation to PD risk, they do suggest that the two loci may have small independent and opposing effects on determining the age of onset of the symptoms. While initial regression analyses in the PPMI cohort showed that age of onset was significantly delayed by carriage of the S/S *‘523’* genotype, this was not replicated after correction for covariates, or in the Australian cohort. However, it is possible that this difference is caused by a country- or geographical-specific effect, as seen in other genetic studies^43^. It is worth noting that only one prior study has examined this association^27^, though it is not clear whether this study completed rigorous analysis of the interactive effects of *TOMM40* and *APOE,* as was conducted in the current study. As such, further in-depth analysis in larger PD cohorts is required to determine the significance of the present findings.

Currently, the functional effects of variation in *‘523’* allele length are poorly understood due to a scarcity of research in this area. While several studies have suggested that the VL allele increases *TOMM40* mRNA expression and the S allele represses expression^12,19,20,44^, others have found no significant differences in mRNA levels between S and VL variants^45^. A recent study demonstrated that overexpression of *TOMM40 *in vitro was associated with greater mitochondrial membrane potentials, respiratory rates, spare respiratory capacities, ATP levels, amyloid-beta resistance, and protein uptake^16^. On the other hand, another study observed a correlation between TOM40 protein deficits and enhanced oxidative stress, reduced ATP production and abnormal complex I protein concentrations in the brains of PD patients and in alpha-synuclein overexpressing murine models^21^. As the literature currently stands, further elucidation of the biological consequences of up- or down-regulated *TOMM40* expression is required to give insight into its potential role in PD risk and disease modification.

## Conclusion

Overall, this study aimed to investigate the risk and disease-modifying role of the *TOMM40 ‘523’* variant in two independent PD cohorts. *TOMM40* plays an essential role in mitochondrial import and mitophagy, and the *‘523’* polymorphism has been associated with age of onset of AD and with age-related cognitive decline. While this study clarifies that *TOMM40 ‘523’* is not in itself a predictor of PD risk, it raises the possibility that, as in AD, it may be a genetic marker for the age of symptom onset in PD. Whilst not conclusive, our findings in the large international PPMI cohort suggest that carriage of the S/S *‘523’* genotype may be protective in terms of delaying the age of symptom onset, and may warrant further investigation in other populations. Importantly, the effect of co-carriage of the *APOE* ε4 allele, which appears to have an opposing effect on age of onset, must be considered in future studies. Though not significant in modulating risk of PD, future studies should consider the possible role of *TOMM40 ‘523’*as a determinant of age of symptom onset, and symptom trajectory. Given a phase 3 clinical trial of a therapeutic for the prevention and delay of onset of AD was recently conducted involving participants stratified by *TOMM40 ‘523’*^46^, the findings reported herein are noteworthy, and may allow the design of symptom-focused studies in PD for much-needed improvements in patient outcomes and care.

## Supplementary Information

Supplementary Information

## Data Availability

Any data pertaining to this article and not published within this article may be requested through collaboration.
