# Supplementary material for: The TOMM40 ‘523’ polymorphism in disease risk and age of symptom onset in two independent cohorts of Parkinson’s disease
Source: Sci Rep. 2021 Mar 18;11:6363. doi: 10.1038/s41598-021-85510-0 (PMC7973542; doi:10.1038/s41598-021-85510-0)
Supplement: Supplementary file 1 — Supplementary Information [file 41598_2021_85510_MOESM1_ESM.docx]

**The *TOMM40 ‘523’* polymorphism in disease risk and age of symptom onset in two independent cohorts of Parkinson’s disease**

Megan C. Bakeberg^1,2^, Madison E. Hoes^1^, Anastazja M. Gorecki^1,3^, Frances Theunissen^1,4^, Abigail L. Pfaff^1,4^, Jade E. Kenna^1,2^, Kai Plunkett^1^, Sulev Kõks^1,4^, P. Anthony Akkari^1,2,4^, Frank L. Mastaglia^1,2,4^ and Ryan S. Anderton^1,2,5*^

^1^Perron Institute for Neurological and Translational Science, Nedlands

^2^Centre for Neuromuscular and Neurological Disorders, University of Western Australia, Nedlands, Western Australia, Australia.

^3^School of Biological Sciences, University of Western Australia, Crawley

^4^The Centre for Molecular Medicine and Innovative Therapeutics, Murdoch University, Murdoch, WA, Australia

^5^Institute for Health Research and School of Health Sciences, University of Notre Dame Australia, Fremantle, Western Australia, Australia.

***Author correspondence:** A/Prof Ryan Anderton, School of Health Sciences, University of Notre Dame Australia, 19 Mouat Street, Fremantle, WA 6959, Australia
Tel: *+61 8 9433 0670*; Email: ryan.anderton@nd.edu.au

**SUPPLEMENTARY INFORMATION**

**
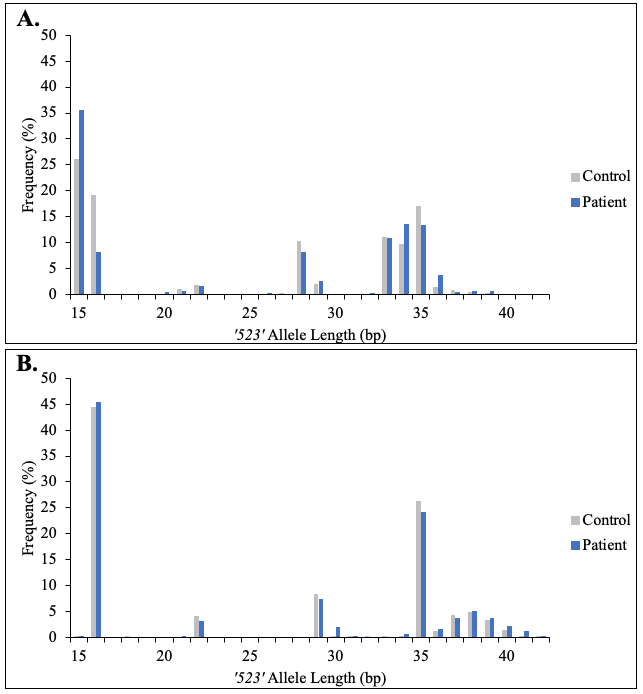
**

**Supplementary Figure 1.** *TOMM40 ‘523’* allele length distributions in the Australian cohort (A) and the PPMI cohort (B). *‘523’, TOMM40* ‘523’ allele; bp, base pairs; PPMI, Parkinson’s Progression Markers Initiative.

**
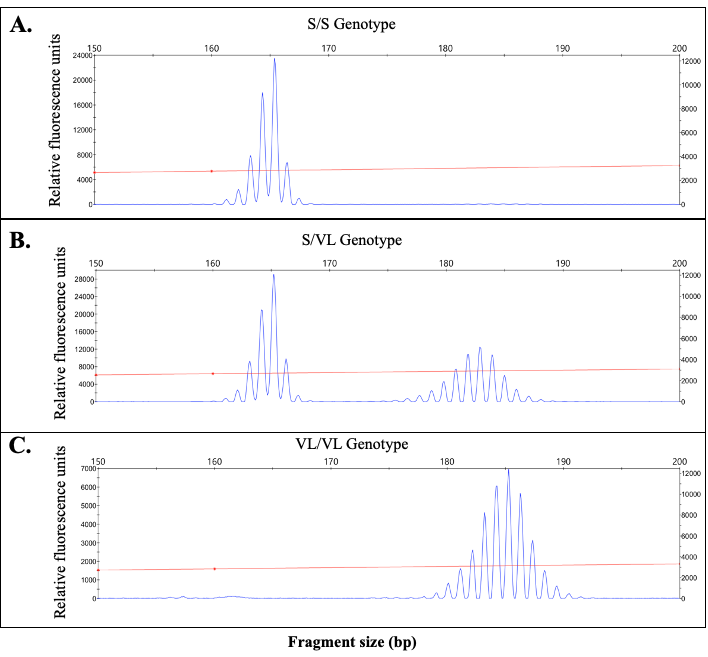
**

**Supplementary Figure 2.** (A) Fragment separation electropherogram for a *TOMM40* ‘*523*’ allele S/VL heterozygote, indicating the highest intensity peak of each allele and the surrounding stutter peaks. To determine each allele’s length (i.e. number of T residues), 150 is subtracted from the size of the highest intensity peak. (B) Fragment separation electropherogram for a *TOMM40* ‘*523*’ allele S/S homozygote. (C) Fragment separation electropherogram for a *TOMM40* ‘*523*’ allele VL/VL homozygote. S/S, short/short; S/VL, short/very long; VL/VL, very long/very long; bp, base pairs.

**
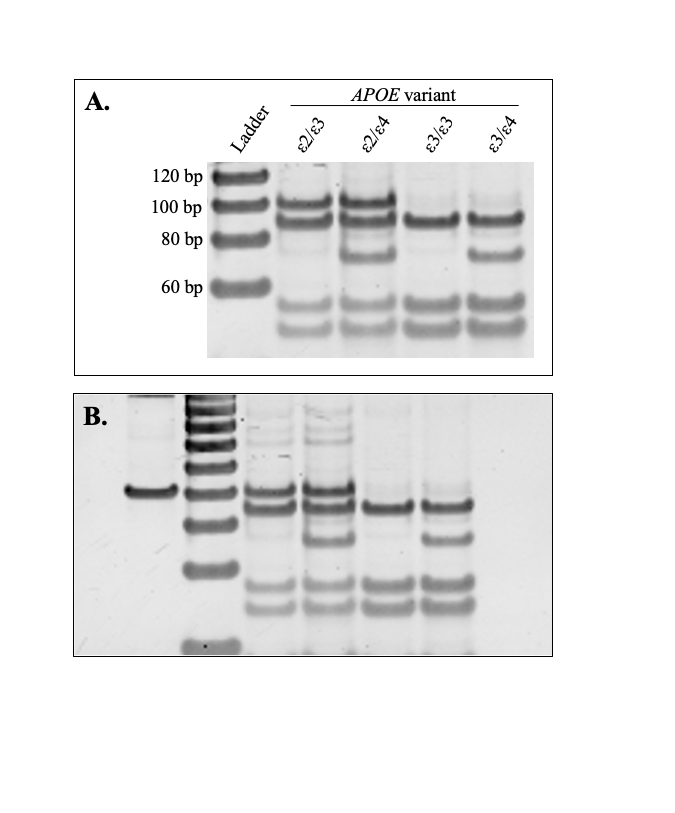
**

**Supplementary Figure 3.** Four RFLP types of *APOE* ε gene digested by HhaL and run on 12% polyacrylamide gels, with cropped image presented (Supplementary Figure 3A). *APOE* ε genotypes labelled above each lane. Full-length blots/gels are presented in Supplementary Figure 3B. Bp; base pairs; ε2/ ε3, Apolipoprotein epsilon 2/epsilon 3 genotype; ε2/ε4, Apolipoprotein epsilon 2/epsilon 4 genotype; ε3/ε3, Apolipoprotein epsilon 3/epsilon 3 genotype; ε3/ε4, Apolipoprotein epsilon 3/epsilon 4 genotype.

**Supplementary Table 1.** Frequencies of *TOMM40 ‘523’* genotype and allele groupings in the Australian and PPMI cohorts.

|  | ***TOMM40* ‘*523*’ Genotype/ Allele** | **Frequency** | | |
| --- | --- | --- | --- | --- |
|  |  | Total  n (%) | Control  n (%) | PD  n (%) |
| *Australian cohort*  *(n=466)* | S/S | 87 (18.7) | 45 (19.5) | 42 (17.9) |
|  | S/L | 52 (11.2) | 27 (11.7) | 25 (10.6) |
|  | S/VL | 187 (40.1) | 91 (39.4) | 96 (40.9) |
|  | L/L | 16 (3.4) | 8 (3.5) | 8 (3.4) |
|  | L/VL | 48 (10.3) | 26 (11.3) | 22 (9.4) |
|  | VL/VL | 76 (16.3) | 34 (14.7) | 42 (17.9) |
|  | S | 413 (44.3) | 208 (45.0) | 205 (43.6) |
|  | L | 132 (14.2) | 69 (14.9) | 63 (13.4) |
|  | VL | 387 (41.5) | 185 (40.0) | 202 (43.0) |
| *PPMI cohort*  *(n=540)* | S/S | 107 (19.9) | 33 (19.2) | 74 (20.1) |
|  | S/L | 58 (10.7) | 18 (10.5) | 40 (10.9) |
|  | S/VL | 213 (39.4) | 68 (39.5) | 145 (39.4) |
|  | L/L | 5 (0.9) | 2 (1.2) | 3 (0.8) |
|  | L/VL | 54 (10.0) | 21 (12.2) | 33 (9.0) |
|  | VL/VL | 103 (19.1) | 30 (17.4) | 73 (19.8) |
|  | S | 485 (44.9) | 152 (44.2) | 333 (45.2) |
|  | L | 122 (11.3) | 43 (12.5) | 79 (10.7) |
|  | VL | 473 (43.8) | 149 (43.3) | 324 (44.0) |
| *Combined cohort*  *(n=1006* | S/S | 194 (19.3) | 78 (19.4) | 116 (19.2) |
|  | S/L | 110 (10.9) | 45 (11.2) | 65 (10.8) |
|  | S/VL | 400 (39.8) | 159 (39.5) | 241 (40.0) |
|  | L/L | 21 (2.1) | 10 (2.5) | 11 (1.8) |
|  | L/VL | 102 (10.1) | 47 (11.7) | 55 (9.1) |
|  | VL/VL | 179 (17.8) | 64 (15.9) | 115 (19.1) |
|  | S | 898 (44.6) | 360 (44.7) | 538 (44.6) |
|  | L | 254 (12.6) | 112 (13.9) | 142 (11.8) |
|  | VL | 860 (42.7) | 334 (41.4) | 526 (43.6) |

Data presented as n (%). PPMI, Parkinson’s Progression Markers Initiative; PD, Parkinson’s disease; S/S, short/short; S/L, short/long; S/VL, short/very long; L/L, long/long; L/VL, long/very long; VL/VL, very long/very long; S, short; L, long; VL, very long.

**Supplementary Table 2.** Frequencies of *APOE* ε genotype and allele groupings in the Australian and PPMI risk association cohorts.

|  | ***APOE*** ε **Genotype/ Allele** | **Frequency** | | |
| --- | --- | --- | --- | --- |
|  |  | Total  n (%) | Control  n (%) | PD  n (%) |
| *Australian cohort*  *(n=434)* | ε2/ε2 | 0 (0.0) | 0 (0.0) | 0 (0.0) |
|  | ε2/ε3 | 43 (9.9) | 20 (8.9) | 23 (11.0) |
|  | ε2/ε4 | 7 (1.6) | 3 (1.3) | 4 (1.9) |
|  | ε3/ε3 | 284 (65.4) | 148 (66.1) | 136 (64.8) |
|  | ε3/ε4 | 84 (19.4) | 46 (20.5) | 38 (18.1) |
|  | ε4/ε4 | 16 (3.7) | 7 (3.1) | 9 (4.3) |
|  | ε2 | 50 (5.8) | 23 (5.1) | 27 (6.4) |
|  | ε3 | 695 (80.1) | 362 (80.8) | 333 (79.3) |
|  | ε4 | 123 (14.2) | 63 (14.1) | 60 (14.3) |
| *PPMI cohort*  *(n=560)* | ε2/ε2 | 4 (0.7) | 3 (1.7) | 1 (0.3) |
|  | ε2/ε3 | 69 (12.3) | 17 (9.6) | 52 (13.6) |
|  | ε2/ε4 | 12 (2.1) | 6 (3.4) | 6 (1.6) |
|  | ε3/ε3 | 340 (60.7) | 111 (62.4) | 229 (59.9) |
|  | ε3/ε4 | 122 (21.8) | 37 (20.8) | 85 (22.3) |
|  | ε4/ε4 | 13 (2.3) | 4 (2.2) | 9 (2.4) |
|  | ε2 | 89 (7.9) | 29 (8.1) | 60 (7.9) |
|  | ε3 | 871 (77.8) | 276 (77.5) | 595 (77.9) |
|  | ε4 | 160 (14.3) | 51 (14.3) | 109 (14.3) |
| *Combined cohort*  *(n=994)* | ε2/ε2 | 4 (0.4) | 3 (0.7) | 1 (0.2) |
|  | ε2/ε3 | 112 (11.3) | 37 (9.2) | 75 (12.7) |
|  | ε2/ε4 | 19 (1.9) | 9 (2.2) | 10 (1.7) |
|  | ε3/ε3 | 624 (62.8) | 259 (64.4) | 365 (61.7) |
|  | ε3/ε4 | 206 (20.7) | 83 (20.6) | 123 (20.8) |
|  | ε4/ε4 | 29 (2.9) | 11 (2.7) | 18 (3.0) |
|  | ε2 | 139 (7.0) | 52 (6.5) | 87 (7.3) |
|  | ε3 | 1566 (78.8) | 638 (79.4) | 928 (78.4) |
|  | ε4 | 283 (14.2) | 114 (14.2) | 169 (14.3) |

Data presented as n (%). PPMI, Parkinson’s Progression Markers Initiative; PD, Parkinson’s disease; ε2/ε2, Apolipoprotein epsilon 2/epsilon 2 genotype; ε2/ε3, Apolipoprotein epsilon 2/epsilon 3 genotype; ε2/ε4, Apolipoprotein epsilon 2/epsilon 4 genotype; ε3/ε3, Apolipoprotein epsilon 3/epsilon 3 genotype; ε3/ε4, Apolipoprotein epsilon 3/epsilon 4 genotype; ε4/ε4, Apolipoprotein epsilon 4/epsilon 4 genotype; ε2, Apolipoprotein epsilon 2 allele; ε3, Apolipoprotein epsilon 3 allele; ε4, Apolipoprotein epsilon 4 allele.

**Supplementary Table 3.** Association between *TOMM40 ‘523’* and PD risk in a subsection of *APOE* ε3/ε3 carriers in the Australian and PPMI cohorts

| *TOMM40* ‘523’ | *Australian cohort*  *(n=284)* | | | | *PPMI cohort*  *(n=302)* | | | | *Combined cohort*  *(n=586)* | | | |
| --- | --- | --- | --- | --- | --- | --- | --- | --- | --- | --- | --- | --- |
|  | Naïve^+^ | | Corrected^#^ | | Naïve^+^ | | Corrected^#^ | | Naïve^+^ | | Corrected^#^ | |
|  | OR (95% CI) | *p* | OR (95% CI) | *p* | OR (95% CI) | *p* | OR (95% CI) | *p* | OR (95% CI) | *p* | OR (95% CI) | *p* |
| S/S | 1.150 (0.677-1.952) | .606 | 1.139 (0.671-1.936) | .629 | 0.906 (0.527-1.557) | .721 | 0.900 (0.523-1.548) | .900 | 1.004 (0.694-1.451) | .984 | 1.000 (0.692-1.446) | 1.000 |
| S/L | 1.505E9 (0.000-0.000) | .999 | 1.460E9 (0.000-0.000) | .999 | 0.000 (0.000-0.000) | 1.000 | 0.000 (0.000-0.000) | 1.000 | 2.789 (0.252-31.035) | .402 | 2.793 (0.251-31.050) | .403 |
| S/VL | 0.859 (0.538-1.370) | .522 | 0.872 (0.546-1.393) | .567 | 1.117 (0.689-1.811) | .654 | 1.118 (0.689-1.812) | .652 | 1.004 (0.723-1.395) | .979 | 1.010 (0.727-1.403) | .952 |
| L/L* | - | - | - | - | - | - | - | - | - | - | - | - |
| L/VL | 0.918 (0.057-14.827) | .952 | 0.935 (0.058-15.188) | .963 | 0.000 (0.000-0.000) | 1.000 | 0.000 (0.000-0.000) | 1.000 | 0.695 (0.063-7.704) | .767 | 0.693 (0.062-7.682) | .765 |
| VL/VL | 0.987 (0.545-1.787) | .966 | 0.974 (0.537-1.764) | .930 | 1.011 (0.562-1.819) | .971 | 1.018 (0.565-1.833) | .953 | 0.968 (0.643-1.457) | .876 | 0.964 (0.640-1.452) | .860 |
| S | 1.016 (0.566-1.825) | .956 | 1.029 (0.573-1.849) | .923 | 1.018 (0.567-1.828) | .953 | 1.010 (0.561-1.817) | .974 | 1.044 (0.696-1.565) | .835 | 1.048 (0.699-1.573) | .820 |
| L | 2.793 (0.287-27.179) | .376 | 2.747 (0.282-26.764) | .384 | 0.000 (0.000-0.000) | .999 | 0.000 (0.000-0.000) | .999 | 1.397 (0.280-6.979) | .684 | 1.392 (0.278-6.962) | .687 |
| VL | 0.814 (0.481-1.377) | .443 | 0.823 (0.486-1.392) | .467 | 1.130 (0.658-1.940) | .658 | 1.139 (0.662-1.959) | .638 | 0.971 (0.673-1.400) | .874 | 0.974 (0.676-1.405) | .890 |

^+^Data taken from Binary Logistic Regression models without correction for covariates.

^#^Data taken from Binary Logistic Regression models with correction for patient sex.

*No *TOMM40 ‘523’* L/L carriers within the subsection of *APOE* ε3/ε3 carriers, in the Australian and PPMI cohorts.

*p* and OR (95% CI) values are calculated for the comparison to all other genotypes or alleles.

PPMI, Parkinson’s Progression Markers Initiative; PD, Parkinson’s disease; OR, Odd’s Ratio*;* CI, confidence interval; *p*, statistical significance (*p* value); S/S, short/short; S/L, short/long; S/VL, short/very long; L/L, long/long; L/VL, long/very long; VL/VL, very long/very long; S, short; L, long; VL, very long.

**Supplementary Table 4.** Frequencies of *APOE* ε and *TOMM40 ‘523’* genotype combination in the Australian and PPMI cohorts.

| *APOE* ε and *TOMM40 ‘523’* genotype combination | Australian cohort | | | | PPMI cohort | | | | Combined cohort | | | |
| --- | --- | --- | --- | --- | --- | --- | --- | --- | --- | --- | --- | --- |
|  | Control | | PD | | Control | | PD | | Control | | PD | |
|  | n | % | n | % | n | % | n | % | n | % | n | % |
| s/s, ε2/ε2 | - | 0.0 | - | 0.0 | 1 | 0.6 | 1 | 0.3 | 1 | 0.3 | 1 | 0.2 |
| s/l, ε2/ε2 | - | 0.0 | - | 0.0 | 1 | 0.6 | - | 0.0 | 1 | 0.3 | - | 0.0 |
| s/vl, ε2/ε2 | - | 0.0 | - | 0.0 | - | 0.0 | - | 0.0 | - | 0.0 | - | 0.0 |
| l/l, ε2/ε2 | - | 0.0 | - | 0.0 | - | 0.0 | - | 0.0 | - | 0.0 | - | 0.0 |
| l/vl, ε2/ε2 | - | 0.0 | - | 0.0 | 1 | 0.6 | - | 0.0 | 1 | 0.3 | - | 0.0 |
| vl/vl, ε2/ε2 | - | 0.0 | - | 0.0 | - | 0.0 | - | 0.0 | - | 0.0 | - | 0.0 |
| s/s, ε2/ε3 | 2 | 0.9 | 7 | 3.3 | 1 | 0.6 | 7 | 2.1 | 3 | 0.8 | 14 | 2.6 |
| s/l, ε2/ε3 | 1 | 0.4 | - | 0.0 | - | 0.0 | 2 | 0.6 | 1 | 0.3 | 2 | 0.4 |
| s/vl, ε2/ε3 | 11 | 4.9 | 12 | 5.7 | 8 | 5.2 | 23 | 6.8 | 19 | 5.0 | 35 | 6.4 |
| l/l, ε2/ε3 | - | 0.0 | - | 0.0 | - | 0.0 | - | 0.0 | - | 0.0 | - | 0.0 |
| l/vl, ε2/ε3 | - | 0.0 | - | 0.0 | - | 0.0 | 1 | 0.3 | - | 0.0 | 1 | 0.2 |
| vl‎/vl, ε2/ε3 | 6 | 2.7 | 4 | 1.9 | 6 | 3.9 | 13 | 3.9 | 12 | 3.2 | 17 | 3.1 |
| s/s, ε2/ε4 | - | 0.0 | - | 0.0 | - | 0.0 | - | 0.0 | - | 0.0 | - | 0.0 |
| s/l, ε2/ε4 | 1 | 0.4 | 2 | 1.0 | - | 0.0 | 2 | 0.6 | 1 | 0.3 | 4 | 0.7 |
| s/vl, ε2/ε4 | - | 0.0 | - | 0.0 | - | 0.0 | - | 0.0 | - | 0.0 | - | 0.0 |
| l/l, ε2/ε4 | - | 0.0 | - | 0.0 | - | 0.0 | - | 0.0 | - | 0.0 | - | 0.0 |
| l/vl, ε2/ε4 | 2 | 0.9 | 2 | 1.0 | 4 | 2.6 | 3 | 0.9 | 6 | 1.6 | 5 | 0.9 |
| vl/vl, ε2/ε4 | - | 0.0 | - | 0.0 | - | 0.0 | - | 0.0 | - | 0.0 | - | 0.0 |
| s/s, ε3/ε3 | 41 | 18.3 | 34 | 16.2 | 26 | 16.8 | 59 | 17.6 | 67 | 17.7 | 93 | 17.0 |
| s/l, ε3/ε3 | 2 | 0.9 | - | 0.0 | - | 0.0 | 1 | 0.3 | 2 | 0.5 | 1 | 0.2 |
| s/vl, ε3/ε3 | 76 | 33.9 | 75 | 35.7 | 50 | 32.3 | 100 | 29.8 | 126 | 33.2 | 175 | 32.1 |
| l/l, ε3/ε3 | - | 0.0 | - | 0.0 | - | 0.0 | - | 0.0 | - | 0.0 | - | 0.0 |
| l/vl, ε3/ε3 | 1 | 0.4 | 1 | 0.5 | - | 0.0 | 1 | 0.3 | 1 | 0.3 | 2 | 0.4 |
| vl‎/vl, ε3/ε3 | 28 | 12.5 | 26 | 12.4 | 21 | 13.5 | 44 | 13.1 | 49 | 12.9 | 70 | 12.8 |
| s/s, ε3/ε4 | 1 | 0.4 | - | 0.0 | 1 | 0.6 | - | 0.0 | 2 | 0.5 | - | 0.0 |
| s/l, ε3/ε4 | 23 | 10.3 | 20 | 9.5 | 17 | 11.0 | 33 | 9.8 | 40 | 10.6 | 53 | 9.7 |
| s/vl, ε3/ε4 | - | 0.0 | - | 0.0 | 2 | 1.3 | 10 | 3.0 | 2 | 0.5 | 10 | 1.8 |
| l/l, ε3/ε4 | 1 | 0.4 | - | 0.0 | - | 0.0 | - | 0.0 | 1 | 0.3 | - | 0.0 |
| l/vl, ε3/ε4 | 21 | 9.4 | 17 | 8.1 | 11 | 7.1 | 22 | 6.5 | 32 | 8.4 | 39 | 7.1 |
| vl‎/vl, ε3/ε4 | - | 0.0 | 1 | 0.5 | 1 | 0.6 | 8 | 2.4 | 1 | 0.3 | 9 | 1.6 |
| s/s, ε4/ε4 | - | 0.0 | - | 0.0 | - | 0.0 | - | 0.0 | - | 0.0 | - | 0.0 |
| s/l, ε4/ε4 | - | 0.0 | 1 | 0.5 | - | 0.0 | - | 0.0 | - | 0.0 | 1 | 0.2 |
| s/vl, ε4/ε4 | - | 0.0 | - | 0.0 | - | 0.0 | - | 0.0 | - | 0.0 | - | 0.0 |
| l/l, ε4/ε4 | 6 | 2.7 | 8 | 3.8 | 2 | 1.3 | 2 | 0.6 | 8 | 2.1 | 10 | 1.8 |
| l/vl, ε4/ε4 | 1 | 0.4 | - | 0.0 | 2 | 1.3 | 4 | 1.2 | 3 | 0.8 | 4 | 0.7 |
| vl/vl, ε4/ε4 | - | 0.0 | - | 0.0 | - | 0.0 | - | 0.0 | - | 0.0 | - | 0.0 |

PD, Parkinson’s disease; S/S, short/short; S/L, short/long; S/VL, short/very long; L/L, long/long; L/VL, long/very long; VL/VL, very long/very long; S, short; L, long; VL, very long; ε2/ε2, Apolipoprotein epsilon 2/epsilon 2 genotype; ε2/ε3, Apolipoprotein epsilon 2/epsilon 3 genotype; ε2/ε4, Apolipoprotein epsilon 2/epsilon 4 genotype; ε3/ε3, Apolipoprotein epsilon 3/epsilon 3 genotype; ε3/ε4, Apolipoprotein epsilon 3/epsilon 4 genotype; ε4/ε4, Apolipoprotein epsilon 4/epsilon 4 genotype.

**Supplementary Table 5.** Generalised linear model investigating association between *TOMM40 ‘523’* and age of PD symptom onset in a subsection of *APOE* ε3/ε3 carriers in the Australian and PPMI cohorts.

| *TOMM40* ‘*523*’ | *Australian cohort*  *(n=136)* | | | | *PPMI cohort*  *(n=205)* | | | | *Combined cohort*  *(n=341)* | | | |
| --- | --- | --- | --- | --- | --- | --- | --- | --- | --- | --- | --- | --- |
|  | Naïve^+^ | | Corrected^#^ | | Naïve^+^ | | Corrected^#^ | | Naïve^+^ | | Corrected^#^ | |
|  | β-CoE | *p* | β-CoE | *p* | β-CoE | *p* | β-CoE | *p* | β-CoE | *p* | β-CoE | *p* |
| S/S | -1.929 | .359 | -1.912 | .363 | 1.757 | .250 | 1.474 | .333 | 0.495 | .694 | 0.392 | .755 |
| S/L^ | - | - | - | - | 9.071 | .361 | 8.151 | .409 | 10.272 | .320 | 9.827 | .341 |
| S/VL | 1.769 | .336 | 1.778 | .334 | -1.381 | .318 | -1.235 | .369 | -0.332 | .767 | -0.295 | .793 |
| L/L* | - | - | - | - | - | - | - | - | - | - | - | - |
| L/VL | -7.970 | .454 | -7.759 | .467 | 2.219 | .823 | 3.995 | .687 | -3.192 | .663 | -2.992 | .682 |
| VL/VL | -0.087 | .971 | -0.136 | .954 | -0.416 | .806 | -0.297 | .859 | -0.166 | .905 | -0.095 | .946 |
| S | 0.462 | .842 | 0.498 | .830 | 0.346 | .836 | 0.180 | .914 | 0.276 | .841 | 0.199 | .885 |
| L | -7.970 | .454 | -7.759 | .467 | 5.673 | .421 | 6.080 | .384 | 1.310 | .827 | 1.291 | .829 |
| VL | 1.929 | .359 | 1.912 | .363 | -1.953 | .198 | -1.655 | .275 | -0.642 | .608 | -0.534 | .6705 |

^+^Data taken from GLM without correction for covariates.

^#^Data taken from GLMs corrected for patient sex.

^No *TOMM40 ‘523’* S/L carriers within the subsection of *APOE* ε3/ε3 carriers in the Australian cohort.

*No *TOMM40 ‘523’* L/L carriers within the subsection of *APOE* ε3/ε3 carriers, in the Australian and PPMI cohorts.

*p* and β-CoE values are calculated for the comparison to all other genotypes or alleles.

PPMI, Parkinson’s Progression Markers Initiative; PD, Parkinson’s disease; β-CoE*,* β-Coefficient*; p*, statistical significance (*p* value); S/S, short/short; S/L, short/long; S/VL, short/very long; L/L, long/long; L/VL, long/very long; VL/VL, very long/very long; S, short; L, long; VL, very long; GLM, generalised linear model
